# Disability and HIV/AIDS - a systematic review of literature on Africa

**DOI:** 10.1186/1758-2652-12-34

**Published:** 2009-11-13

**Authors:** Jill Hanass-Hancock

**Affiliations:** 1 Health Economics and HIV/AIDS Research Division (HEARD), University of KwaZulu-Natal, South Africa

## Abstract

This systematic review focuses on empirical work on disability and HIV/AIDS in Africa in the past decade and considers all the literature currently accessible. The review presents data from different surveys and summarizes the findings. In this way, it convincingly reveals that people with disabilities are very vulnerable to contracting HIV, and lack access to information, testing and treatment. The review further reveals gaps in the research and areas of concern. While vulnerability and accessibility have been investigated, there are few prevalence studies or evaluations available. A certain amount of work has focused on the deaf population, but little has been done for other disability groups. A growing area of concern is sexual abuse and exploitation of people with disabilities. Only a few studies or interventions focus on this crucial area.

## Background

The year 2008 was a special one. It was during this year that the XVII International AIDS Conference (AIDS 2008) in Mexico City, as well as the 15^th ^International Conference on AIDS and STIs inAfrica (ICASA 2008) in Dakar, Senegal, focused on disability as an issue in HIV/AIDS. Yet there are still many complaints about the paucity or lack of research [1-3]. While it may be true that there is still not enough research on HIV/AIDS and disability available, it is also true that mainstream research is sometimes ignorant about or sceptical towards the importance of disability in the HIV/AIDS field. It is, however, incorrect to say that there is no research available.

The field of disability in HIV/AIDS has been growing in recent years. Because research is scattered, there is a need to consolidate the available literature in a systematic way. There is a tremendous need for knowledge distribution on the topic as advocates often need "hard data" to convince health officials of the need to act on disability issues; the latter is particularly true for Africa.

This review was initiated as a result of the needs of African disabled people's organizations and particularly, as a result of discussions with the Disability HIV and AIDS Trust. It attempts to describe the state of empirical research in Africa and systematically brings together all the available literature. Other reviews on literature about HIV/AIDS and disability have been published [4]. The presented review differs from others in the sense that it looks at the whole of Africa, is systematic and therefore includes more studies than previous reports. It is predominantly descriptive.

## Methods

A systematic literature review of HIV/AIDS and disability in Africa was conducted for all studies available up until 31 December 2008. The review focuses on papers related to people with disabilities and their vulnerability to HIV. It aims to bring together scientific papers on the topic, describe the content, summarize it, and identify gaps for further research. Disability was defined in accordance with the World Health Organization (WHO) definition as "a complex phenomenon that manifests itself at the body, person or social level. According to this model, these three dimensions of disability are outcomes of interactions between health conditions, other intrinsic features of the individual and extrinsic features of the social and physical environment" [5].

Nevertheless, people do not necessarily publish disability-related work in WHO terms. Medical synonyms therefore had to be identified during the search process to track down all available documents. Mental health was not included in this review because such studies often look at mental health problems as a result of HIV infection or it is difficult to determine if the mental health condition or the HIV infection occurred first. This is a limitation of this review.

### Search Strategy

Altogether, 24 electronic databases, which were relevant either to HIV/AIDS or disability, were searched. The data was collected between June 2008 and December 2008 (final date: 31 December 2008) from the following databases: ADOLEC, AIDSLINE, AIM, AJOL, Anthropology Index, Cambridge Online Journals, Cochrane Library, CSA Illumine, EBSCOhost, iLink OPAC, ISAP, OCLC, NIPAD, ProQuest, SAbinet, ScienceDirect, the Web of Science Social Science Citation Index, Wilson Web Education, Wilson Web Humanities, PubMed, the Quarterly Index of African Periodical Literature, Psychology Journals, the Social Science Citation Index, SOURCE and the UKZN Federated Search.

In the initial search, no restrictions were applied in terms of age, country, disability group, gender, geography, economic characteristics, outcome measures or empirical approach. Libraries of agencies involved in disability work (Disabled People International, Africa Campaign) and relevant conference documents from 2000 onwards (AIDS 2008, ICASA 2008 and two international symposia focusing on HIV/AIDS and disability) were also searched. Unpublished and ongoing research was accessed through contacting activists or researchers, who were approached either through the Health Economics and HIV/AIDS Research Division (HEARD) disability and HIV network, or through the African Campaign on Disability and HIV/AIDS.

Because the search contained no restrictions other than that the literature had to focus on HIV/AIDS (problem) and disability (population), it was possible to include studies which focus predominantly on related issues, for example, reproductive health, as long as these studies included HIV in the research design. Search terms were identified through MeSH, and these were used to create the search string. Common synonyms for HIV, AIDS, and disability and its different forms, were identified and then entered into various search engines (see appendix 1).

### Exclusion criteria

Because the main purpose of this review was to focus on established findings on disability and HIV/AIDS, only papers that included both phenomena were selected. A geographical filter was brought in at a later stage and only studies conducted in Africa were included in the sample. To bring in the filter earlier might have excluded those studies that did not indicate the geographical focus in their keywords.

**Figure 1:**
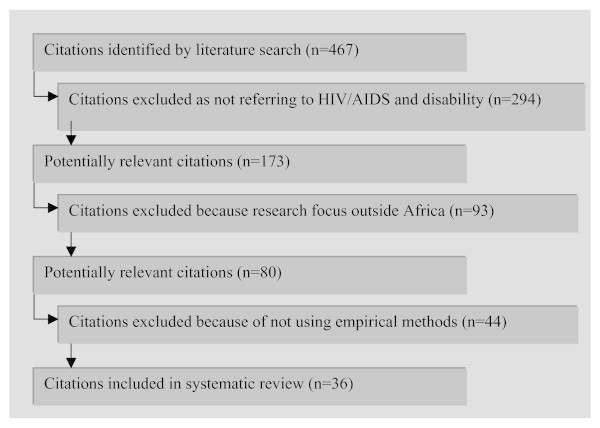
**Included and excluded citations in systematic review**.

The final sample included only studies that made use of empirical methods. Anecdotal stories were excluded from the sample (see figure 1).

### Data extraction and analysis

The final sample was analysed with regard to research methods used, geographical distribution, targeted population (disability type, gender and age), and research outcomes. Excel was used to assist the analysis. As results of data focused on very different areas within the field, it was not possible to determine a statistical meta-analysis. Instead, results were used for content analysis. Some of the available research has not been published. The study can therefore not make any assumptions about possible bias within individual research papers.

## Results

The search retrieved 467 records, of which 80 were potentially relevant citations. After excluding writings that did not have an empirical approach, 36 studies were used in the final sample (see figure 1). Eighteen of those were published in peer-reviewed journals or presented at international conferences, while the others were reports retrieved from organizations or government departments.

### Description of studies

**Figure 2:**
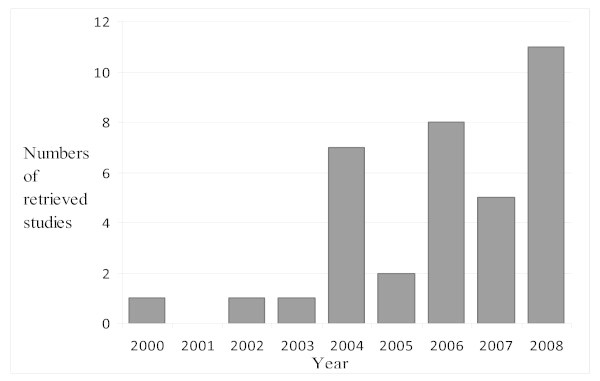
**Number of publications on HIV/AIDS and disability per year**.

Before 2004, almost no research exists that focuses on HIV/AIDS and disability in Africa (see figure 2). In 2000, Osowhole conducted the first cross-sectional study that also used in-depth interviews [6]. In 2003, data from Uganda [7] became available in a study which used both quantitative and qualitative methods. In 2004, publications increased as data from the World Bank/Yale University global survey as well as from Zimbabwe, Malawi, Kenya and Ethiopia, became available [8-14].

In the past few years, the sector has received more attention and more research has been published. It is very likely that even more work has been produced, for example, in support of government or non-governmental organizations' work. Reports from such surveys are not always published and are therefore difficult to access. Such reports might not be included in this review.

The studies used a variety of methods. Fourteen studies approached the field with a qualitative approach, while seven studies used quantitative methods. Ten studies mixed their research design, using qualitative and quantitative methods. Four studies focused predominantly on literature and policy reviews, some adding a few in-depth interviews (see Table 1). Studies were of various sizes and used between seven and 3358 participants. The largest sample size came from an operational research in Kenya with 3358 deaf and hearing participants [15]. While most studies focused directly on HIV/AIDS and disability, two studies were part of a more comprehensive study on reproductive health [7,8]. One study accessed sexual abuse and its links to HIV [16,17] and one study inquired into the social construction of disability and its links to HIV [18].

**Table 1:** Research methods used by different studies

Quantitative approaches	- 11 cross-sectional studies
	- 7 KAP surveys
	- 6 rapid assessments
	- 2 prevalence studies
	- No study uses national data (e.g., DHS)
Qualitative approaches	- 19 in-depth interviews
	- 14 focus groups
	- 5 case studies

Reviews	- 4 document or policy reviews (content analysis)

Only four studies used control groups [15,19,20]. Two of these used random sampling [19,20]. Besides this, very few studies give detailed information about their sampling methods.

Most studies used either a cross-sectional approach or knowledge, attitude and practice (KAP) surveys (see table 1). Some surveys were also labelled as cross sectional when, in reality, they measured only one component, e.g., knowledge. Most studies concentrated only on a small area or a school and are therefore not representative of the larger context. However, some studies have a remarkable sample size. For example, the Kenyan study from Taegtmayer *et al *[15] had 3358 participants, half of which were deaf. The Kenyan study from Handicap International had 618 participants and the Zimbabwean study from Centre for Approved Social Science (CASS) had 669 participants [21,22]. In the latter case, participants were exclusively people with disabilities and their caregivers.

Often, the qualitative analysis is not underpinned by theory or at least described in the literature. Only two studies worked with social theory throughout their work [18,23]. Many studies mention the social model of disability, but fall short when applying this to the research design. Medical terms, like deafness and blindness, are often used instead of the more encompassing social concepts of disability. It is therefore not surprising that other impairments, such as albinism or epilepsy, are only peripherally discussed. However, in some African contexts, these might well be considered disabilities due to social stigma [5,24-26]. This is a particular area of concern since many studies argue that disability is a social phenomenon and should be seen in this light.

**Figure 3:**
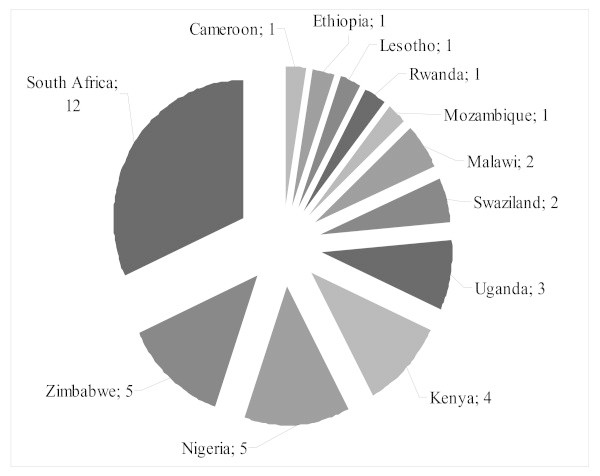
**Distribution of studies focusing on HIV/AIDS and disability in Africa**.

Geographically, most studies focus on southern and eastern Africa (see figure 3): South Africa and Zimbabwe in the south, and Uganda and Kenya in the east. Twelve studies were conducted in South Africa, which is a third of all the studies included in the sample, yet only a few of these used quantitative methods. Nigeria has produced the most research in the western part of Africa.

The most comprehensive study, which included sexual reproductive health, employment and questions related to HIV/AIDS, was carried out in Malawi [8]. It included questions about HIV knowledge, sexual behaviour and history, stigma, perceived risk to HIV infection, sources of HIV information, channels of communication, and accessibility of health services.

Comparatively little research has been conducted in countries in the western part of Africa, yet most, and one of the first, surveys come from Nigeria [6]. One of the two available prevalence studies comes from Cameroon [27]. The second prevalence study comes from Kenya [15].

**Figure 4:**
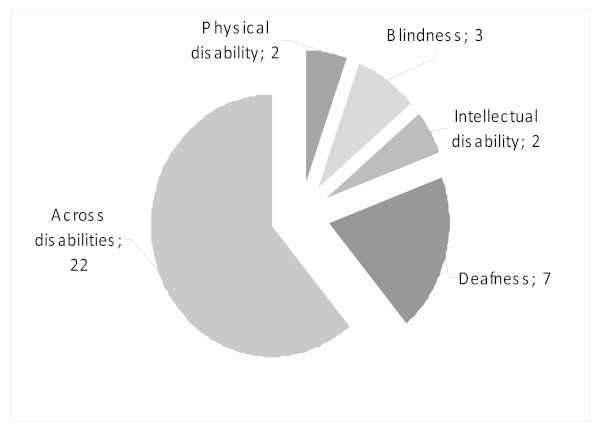
**Population focus of studies**.

Most studies [7,8,14,18,21,23,28-36] conducted investigation across disabilities, but some of them produced disability-specific information from questionnaires designed to capture the latter. Out of the different disability groups, most attention was given to deafness, with seven studies focusing exclusively on the issue [6,19,20,22,27,37,38]. Very few studies focused on physical disability [23,39-41], intellectual disability [16,17,42] or blindness [43-45], and none focused on other issues (see figure 4).

As already mentioned, no study focuses on phenomena like albinism or on abnormalities that are not classified as impairments. Albinism is randomly included in some studies that look across disabilities. Out of the 36 studies, 19 focused on adults, 10 on children or youth, and four included gender as a major component. Others were desk studies including a few key stakeholder interviews. No study focused on sexual orientation, for example, homosexuality, disability and HIV/AIDS.

Thematically, most studies focused either on vulnerability (20 studies or 54%), or on access to HIV prevention and AIDS treatment (18 studies or 48%). Seven studies (18%) looked at cultural issues, disability and HIV/AIDS, and six (16%) included sexual abuse and six included sexuality in their study design. Only one study focused on sexual self-esteem and body image [23], two studies evaluated an intervention [6,17], and two prevalence studies are available [15,27].

### Description of outcomes

Studies revealed that people with disabilities, with some exceptions [46], are aware of HIV in most countries [6,10,11,20] and perceive themselves as particularly vulnerable to contracting HIV. The Ugandan survey, in which 371 people with disabilities participated, revealed that 55% of people with disabilities perceived themselves as at risk of contracting HIV [7]. Similarly, Ngazie's study in Zimbabwe, with 67 participants in an urban area, showed that 75% of participants perceived themselves to be at risk [29]. In a Kenyan study of 1709 deaf people, 80% perceived themselves to be at risk [37], and in South Africa, 93% of the 15 blind participants in Phillander's study [44] indicated that they could be at risk of contracting HIV.

The two prevalence studies, one from Kenya and one from Cameroon, indicate that the prevalence rate for deaf people is as high as the prevalence rate for their non-disabled peers, with 7% prevalence in Kenya (6.7% in the hearing population) [47] and 4.4% (5% national average) in Cameroon [27]. There are no data available on other disability groups.

Most studies revealed that people with disabilities experience barriers to prevention, interventions and treatment [7,12,14,18,36,43,44,46,48]. Special schools are excluded from prevention campaigns or lack sex education [16,18,41,49]. Clinics are physically inaccessible, and transport unaffordable or not suitable for wheelchair users [35,43,46].

For people with sensorial impairments, certain channels of communication are inaccessible. Otte *et al *[43] in Nigeria report that visually impaired people experience hospitals and billboards as inaccessible, and the Phillander and Swarts participants emphasize that Braille and audiotapes are necessary to make AIDS services accessible to people with visual impairments [44].

Other studies reveal that volunteer counselling and testing staff, practitioners, nurses and police officers are not able to communicate with deaf people [18,47,50] and confidentiality is therefore often compromised. In addition, the social construction of disability marginalizes and stigmatizes people with disabilities. In this context, professionals might treat people with disabilities with insufficient respect or simply forget about them as they falsely believe that these people are asexual [18,35,36,40,41,48].

Surveys, not surprisingly, reveal that people with disabilities have less knowledge about HIV than other people. Part of the world survey provided data indicating that deaf participants are more likely to believe in incorrect modes of transmission (p < 0.05), like kissing, hugging, touching or sharing dishes [19,51]. The two studies conducted in Nigeria and Swaziland used comparison groups. Otte *et al*, whose research included a comparison group [43], reveal similar data about blind adolescents in Nigeria. The study found that blind adolescents are prone to believing in wrong modes of transmission and prevention (p = 0.001). However, the same study found no significant differences for questions related to HIV treatment [43].

Wazakili *et al *(no comparison group) make similar claims about young people with physical disabilities. Their study reveals that the participants have limited factual HIV knowledge and that their choices about sexual behaviour are not informed by what they know. The authors emphasize that the sexual behaviour of adolescents with disabilities is particularly influenced by their living contexts [40,41]. Looking at disability more broadly, Munthalie's study in Malawi (no comparison group) yields similar results and states that "knowledge about HIV is basic". Thirty-six percent of the respondents stated that HIV is AIDS, and 42.5% said that they could tell if someone has AIDS "by just looking"[8].

Giros's study in Kenya (no comparison) reveals that although 86% of the deaf respondents are aware of HIV/AIDS and its transmission, some still believe in false modes of infection: "41% named biting of mosquitoes, kissing (39.6%), and sharing of eating and drinking utensils with HIV positive persons (26.4%) as possible ways of transmission" [11]. Disability and Development Partners' study in Mozambique (no comparison group) mirrors these findings: 84% of the respondents answered that they do not know what HIV or AIDS is, and 70% answered that they do not know how HIV is transmitted [8].

Unfortunately, not one of the four studies is compared to the general population and we therefore do not know if this misinformation applies specifically to the disabled population or if it is a common phenomenon in the cultural context. At least, one can conclude that people with disabilities are as misinformed as the rest of the population and to reach them, prevention needs to become accessible.

On a similar note, Dawood's study (no comparison group) in a Durban school (South Africa) shows that youngsters with moderate learning difficulties (the authors speak of mild mental retardation) [42] have "critical gaps and erroneous beliefs regarding knowledge of HIV/AIDS", with one in five learners "subscribing to mythical beliefs in cure" (such as sex with a virgin), and one in five believing that there is a cure for AIDS or believing in "erroneous ways of transmission (e.g., transmission through insects or non-sexual contact)" [42]. Other studies reveal that teachers of children with intellectual or learning disabilities might not feel able or willing to teach these youngsters about HIV and sexuality as they "don't want to wake sleeping dogs"[18].

In spite of popular misconceptions, people with disabilities (PWD) are in fact sexually active. Focusing on adults with disability, the Malawian study (341 PWD) revealed that 76% had been sexually active [8], while in Cameroon, 80% (126 deaf people) [27] and in Kenya, 89% (1706 deaf people) [37] of the participants indicated that they had had sex. Pregnancy rates also indicate sexual activity and as a Ugandan study (371 PWD) showed, 77% of the participating women had been pregnant before [7].

Focusing on adolescents, the Kenyan study revealed that 29% of people with disabilities had had sex before the age of 16, while a South African study (90 adolescents with intellectual disability) showed that 17% were involved in sexual activity between the ages of 14 and 16 [42]. Contrary to common misconceptions, sexual debut might even occur earlier. Touko's study [27] (126 deaf participants) revealed that the average age for first sexual encounters in deaf people in Cameroon was about a year earlier than the national average (16.5 years). Unfortunately, there are no more quantitative data available on sexual behaviour of people with disabilities in Africa in the context of HIV.

Another growing area of concern is sexual abuse and exploitation of people with disabilities. Munhalie's study in Malawi (341 PWD) revealed that 17% of the participants were forced into their first sexual encounter [8], while 7% of Kenyan (1704 deaf people) [37] and 22% of Ugandan participants (371 PWD) indicated abuse at first sexual encounters [7]. In an Ethiopian study (250 PWD), which focused on sexual violence, 46.4% of the participants reported that they had been victims of sexual violence during their lifetime [52], with most of the victims being women between the ages of 19 and 29. In 70% of the cases, disability contributed to the assault. Similarly, in the focus group discussions of Yousafzai *et al*, sexual exploitation and abuse were believed to be higher among disabled women than their non-disabled peers because the former are perceived to be "free" from the HIV virus [13].

Although sexual abuse is a reality for many people with disabilities in Africa, only a few cases are reported. In the Ethiopian study [52], few cases were reported to the police (18%), because people fear that they will not be believed or that they will be subjected to further victimization. Similar reasons for non-disclosure were given in other studies [7,16-18,21]. Participants in Phillander and Swartz's study (80% of 15 blind people) believe that economic dependency, in particular, contributes to gender-based violence [44].

Economic dependency has been described in many different studies outside AIDS research and certainly is an issue for people with disabilities. Sexual exploitation is occuring in this context. As described by Hanass-Hancock [53], people with disabilities, and in particular, people with intellectual disabilities, might use sexuality as a means to prove that they are capable of having sex and having children, or to gain love and recognition, even if it is only for a short time. This can easily be exploited, lead to unprotected sex, and increase the risk of HIV infection.

Phillander's and Swartz's study [44] reveals that 93% of the visually impaired participants believe that "the general public holds myths about people with visual impairments, including beliefs about asexuality or abstinence"; 20% of the participants gave an indication that there are some people who believe that sex with a virgin or a disabled person can cure AIDS. This phenomenon, described by Groce as "virgin cleansing" [54], has been reported in other studies as well [10,13,18,55-57]. These reports usually come from people with disabilities, not from the perpetrators or the victims. It is therefore difficult to make assumptions about how widespread this practice is in the southern African context.

Participants in various studies indicated that people with hearing impairments are soft targets for sexual assault since it is believed that they cannot shout for help or talk about the abuse [53]. Similarly, children with disabilities, particularly severe disabilities, are regarded as at risk of sexual abuse [16,17,21]. Parents of children with disabilities might therefore be overprotective of the children, which often leads to isolation [21,55,56].

Two of the main problems in sexual abuse cases are the lack of services available to people with disabilities [12,21] and the fact that services are not sensitive to disability issues. The CASS study in Zimbabwe emphasises that there is no disability-sensitive evidence gathering in the judicial system [21].

Dickman *et al *describe similar problems in the judicial system in a study they conducted on rape trails (n = 94) of people with intellectual disabilities in Cape Town, South Africa [16,17]. The study reveals that 94% of the cases were females. In 88% of the cases, the complainant knew the accused, and in 12% of the cases, more than one accused was involved. Dickman *et al *also describe an intervention of the Cape Mental Health [16,17], which specializes in rape cases of people with intellectual disabilities. The study by Dickman *et al *is the only one available that assesses the judicial response to rape of people with disabilities in Africa [17].

Other cultural aspects, such as polygamy, wife sharing, and gender imbalances while negotiating safer sex [7,44], are also mentioned as factors that increase the risk for people with disabilities because they are often seen as less worthy than others. People with disabilities are more likely to be chosen as the second wife, additional partner or for an affair only [10]. This applies particularly to women [53]. Multiple partnerships and unprotected sex therefore become more likely, which in turn increases infection risks. Touko's study (126 deaf people) in Cameroon revealed quantitative data to support this thesis. In this study, 45% of the participants indicated that they were engaged in multiple relationships, a figure above the national average [27].

Even mothers of children with disabilities can be affected by the negative stigma of disability and the constraints put upon a family. A Zimbabwean study (67 parents of children with disability) showed that in 60% of the cases, the father left the family as soon as a disabled child was born. Parents who give birth to a disabled child might also hurry to produce another child to prove that they are not responsible for the disability or to provide a playmate for the disabled child who will be excluded by other community members [29]. Recent data [21] and desperate calls for help show that orphans with disabilities in Zimbabwe are particularly affected by HIV and that an urgent need for action exists.

With regard to voluntary counselling and testing, three studies produced data. While the testing uptake in Kenya of 53% is relatively high [37], surveys in Uganda (371 PWD) [7] and Malawi (341 PWD) [8] reveal a very low uptake of 6% and 10%, respectively. The Malawian study, in addition, shows that only 42% of the participants knew how to use a condom. The condom uptake in general was also very low.

While Touko's study in Cameroon (126 deaf participants) indicates that about 47% of deaf people used condoms the last time they had sex, a study in Uganda reveals that only 24% of men and 10% of women with disabilities use condoms. As a result, not only HIV, but also sexual transmitted infections (STIs), have become a problem. Only 38% of PWD who suffered from STIs in Uganda were treated. Not surprisingly, knowledge about STIs is not well distributed in any of the countries. The Cameroon studies reveal that only 50% of the participants knew about sexually transmitted infections.

Many studies conclude that disability-specific HIV/AIDS prevention programmes and interventions need to be designed, and mainstream services should be made accessible for people with disabilities [9,12,44,48,58]. Mobilization of, for example, deaf peers showed to be instrumental in gaining confidence to participate in voluntary counselling and testing [27]. The effectiveness of channels to disseminate knowledge depends on area-specific circumstances and is disability specific. In Malawi, which is largely a rural area, the radio is the most used source of information (except for deaf people) [8], while in the Durban metropolis, pupils with moderate learning disabilities gather information predominantly from television [42]. Further data on this issue are not available.

## Discussion

In this review, 36 studies approaching a total of 7759 participants, were reviewed. Research is particularly evident around HIV knowledge. It is often argued that people with disabilities have less knowledge about HIV as they have less access to HIV information and interventions. Studies which have used comparison groups can successfully argue this case. This argument becomes even stronger with the recently released national South African study [59] indicating that people with disabilities have the lowest HIV knowledge of all the assessed groups. More detail about knowledge indicators are not available in the report.

Studies that provide us with such details show, however, that HIV knowledge is lacking in areas of HIV transmission and prevention. Often HIV risk through sexual transmission is known to the participants, while other modes of transmission, such as mosquito bites or hugging and kissing, achieve very low scores. Other studies that don't use comparison groups indicate a similar scenario, but cannot tell us if the lack of knowledge is due to disability or the particular cultural context. Furthermore, it is debatable if the lack of HIV knowledge in relation to some modes of transmission, such as hugging, kissing and mosquito bites, can really explain the risk of exposure to HIV infection for people with disability when at the same time, results indicate that they know about the risk through sexual transmission.

The reviewed studies indicate, however, that people with disabilities are a vulnerable group due to a number of factors, some of which have been mentioned in the world survey on HIV/AIDS and disability, conducted by Groce [9]. People with disabilities are seen as vulnerable as they:

• Are more likely to believe in wrong modes of transmission

• Are less likely to receive information and resources to ensure "safer sex" because common prevention programmes do not include disability-specific approaches

• Are more likely to be excluded from or deprived of education, particularly sex education

• Are at increased risk of violence and rape and are also without legal protection

• Are, as children, particularly vulnerable because parents (in particular, fathers) might desert children

• Have less access to testing and treatment because transport and medication might be unaffordable, clinics might not be not accessible, voluntary counselling and testing might not be disability specific, or counselling may violate basic requirements of confidentiality

• Are marginalized, and the double stigma of disability plus HIV/AIDS might make it difficult to disclose HIV status, particularly in the case of women who depend on their families, friends, boyfriends or husbands.

### Shortcomings and needed data

In recent years, awareness of disability has changed in some countries, and some pilot projects have shown how to include people with disabilities in voluntary counselling and testing, as well as in AIDS treatment [36,60]. Yet when it comes to sex education, condom use and sexual abuse, there is little evidence of effort. The data show that although people with disabilities are aware of HIV, they lack HIV knowledge, and within the disabled group, condom use is still low. While the latter might be caused through power imbalances in sexual relationships, we must take note that, according to some studies, about half of participants indicate that they do not know how to use a condom.

The persistence of myths about transmission within the disabled community also indicates that sex education is not consistent and lacks information about disability-specific interventions. As only one prevention intervention for people with disabilities has been evaluated and only three studies have focused on schools, it is not possible to decide what exactly is lacking in the education system. One can only draw conclusions from writings and comments in the field, such as those made by Wazakili, who pushes for more holistic education for people with physical disability and the inclusion of sex education, in particular [41].

In general, sex education in Africa is often dominated by abstinence messages [61], which might be detrimental to people with disabilities, who at times may need special intervention, demonstrations and explanations that go beyond conservative imagination [35]. A blind person might need to touch and feel, a deaf person needs signs, and a person with intellectual disabilities needs plain and direct instruction with pictures that leave no room for false interpretations. This might become very uncomfortable for teachers who most likely need support themselves to perform this special task [62].

Beside scarce research on prevention intervention, no data are available on factors that influence sexual behaviour, for example, substance abuse, peer pressure and migration. The complex combination of being disabled and homosexual has also not been mentioned in any of the studies, although evidence from other countries suggest that this is a particular challenge [63]. Given the fact that homosexuality in Africa is at best taboo, and in some countries, even criminalized, this might be an area that needs further exploration.

Issues of drug abuse, homosexuality, peer pressure and migration have been investigated in mainstream HIV research [64], but not in the field of disability and HIV/AIDS in Africa. In addition, only one study focuses on body image and self-esteem, something that could be influential in sexual behaviour and the subsequent risk to HIV infection and reproductive health. Wazakili's study, for example, indicates that people with physical disabilities do not use their HIV knowledge to make sexual decisions and it would be valuable to find out what does influence them. More research is needed in this area.

The criticism is often that national data collection does not include disability indicators. National prevalence studies should include disability so that prevalence can be assessed. This might be complicated because of a scarcity of sign interpreters to conduct such studies nationally. While this should be the long-term goal, smaller studies can be linked to national data collection and focus on particular areas that are representative. The operational research conducted in Kenyan voluntary counselling and testing centres for the deaf has provided some experience from this field [47]. In addition, mainstream researchers need to be encouraged to include disability indicators similar to gender indicators as a general requirement.

It should be noted that national data are seldom utilised for HIV/AIDS and disability research despite the fact that such surveys as the Demographic Health Survey are available in various African countries. In South Africa, the survey includes disability and HIV/AIDS items, which could be analysed without collecting new data. In addition, the recently released South African national HIV prevalence, incidence, behaviour and communication survey [59] now includes people with disabilities in its sample (survey released after the review dateline). The results show that the prevalence of HIV within the disabled group is 14.1% higher than the national average and also higher than other risk groups, such as men who have sex with men, recreational drug users and high-risk drinkers.

Similarly, Touko's new data from Cameroon [65] indicate double the HIV prevalence rate within the deaf population compared to the national average. On a similar note, a recent study from Collins (2009) in a public psychiatric institution in KwaZulu-Natal, South Africa, also revealed a high HIV prevalence rate among people with mental illnesses, with women being more likely to be infected with HIV than men, a trend also being observed in the general population in southern Africa [66].

These newer sets of data certainly show how to include disability within national surveys or smaller-scale disability-specific studies. The results stress the point of providing interventions for people with disabilities and those with mental conditions.

Disability research needs to be more focused on disability-specific issues. A substantial amount of research focuses on and includes deafness, and some research is available on people with physical, intellectual and visual disabilities. There is an appalling lack of data on people with mental health conditions and their risk of infection with HIV in sub-Saharan Africa. However, data from other geographical areas and a recent study in KwaZulu-Natal indicate that "people with severe mental illnesses have a higher HIV prevalence than the general population" [66,67]. Collins argues in this context that "HIV care and treatment programmes should be made available to people with psychiatric symptoms"[66].

The above-mentioned studies discuss issues of disability with classical medical terms. Far less research has been conducted that consistently applies the social model of disability in the research design [18]. While it might be very difficult to apply the social model of disability in practice, it is not impossible, and experiences from other fields may guide the way here. For instance, in his description of the application of the social model to South African law practice, Ngwena [68,69] discusses how the social model of disability can be applied to the Employment Equity Act.

Because most research focuses on or includes deafness, it is not surprising that a substantial number of interventions concentrate on deafness. Studies like the Kenyan one link deaf people with a higher voluntary counselling and testing uptake rate compared to other disability groups [37]. This result can be regarded as a success in targeting a particular group, but a failure when it comes to providing for others groups.

Most studies, although they do not place emphasis on it, indicate that sexual abuse of people with disabilities is an area of concern. It is clear that more data are needed to galvanize officials into action. People might feel apathetic about the problem, but the Cape Mental Health programme in South Africa shows that effective interventions in the judicial system are in fact possible. Research needs to take such positive examples forward as lessons for other areas.

## Conclusion

People with disabilities are at risk for exposure to HIV infection and are less likely to access prevention, testing and treatment. Research in the area of disability and HIV/AIDS is still scarce, but a growing body of literature is developing. The quality of the research varies, with some studies using high qualitative designs, while others lack basic methodology descriptions, such as sampling procedures. This indicates that besides more research on disability and HIV, capacity building is urgently needed and future research projects need to take this into consideration. Apart from the need for more capacity within Africa, more research is needed in the following areas:

• Prevalence studies

• Operational research (antiretroviral treatment for people with disabilities)

• Evaluation of prevention interventions

• Sexual abuse

• Sexual identity and body image

• Analysis of national data

• Disability and other marginalising attributes (e.g., homosexuality).

While research has produced data to reinforce the argument that people with disabilities are at least as likely to become infected with HIV as their non-disabled peers, some studies stress the point that they are more at risk [9,18]. Recent data on HIV prevalence support this claim in some African countries. We, however, do not yet fully understand why people with disabilities are at higher risk of being exposed to HIV and how this relates to specific contexts within African countries.

The Joint United Nations Programme on HIV/AIDS recently recognised in its policy brief on disability and HIV that people with disabilities are a key group at increased risk of exposure to HIV infection [70]. How general this applies will remain an open-ended argument until more data from prevalence studies are available.

For the African context, however, it has been successfully argued that people with disabilities have been left out of HIV/AIDS prevention and treatment programmes for far too long [70]. However, the exclusion of people with disabilities in Africa is not an isolated phenomenon of HIV and AIDS service delivery. People with disabilities experience barriers while trying to access education and health services in general. Similar to other resource-poor settings, African countries experience challenges to make services accessible. However misconceptions about and ignorance towards disability leads to exclusion as well [18].

While successfully addressing misconceptions might take a little longer because they have to be understood and addressed within African cosmology and interpretations of diseases and misfortunes [71], ignorance can be addressed more rapidly. As described by Groce in 2004, health services can be made accessible for people with disabilities through moving crucial services, such as voluntary counselling and testing to the bottom floor. Mobile clinics could use tents instead of caravans, and information could be made available on tapes if Braille is too expensive. People with disabilities should also be actively involved in service delivery, a fact given emphasis by the disability movement of the African continent [70].

## Competing interests

The author declares that she has no competing interests.

## Authors' contributions

JHH has written this article by herself.

## Appendix 1

### Search string

((hiv OR aids OR (hiv infection*) OR (human immunodeficiency virus) OR (human immunodeficiency virus) OR (human immuno-deficiency virus) OR (acquired immun*) OR (deficiency syndrome) OR (sexually transmitted disease*) OR (sexually transmitted infec*) OR STD* OR HIV/AIDS) AND (PWD OR (people with disability) OR (people with disabilities) OR (person with disability*) OR (children with disabilit*) OR (Orphan* with disabilit*) OR disabilit* OR impairment OR blindness OR blind OR deafness OR deaf OR (physical disabilit*) OR (intellectual disabilit*) OR (mental disabilit*) OR (deaf blind))
